# 3D data-augmentation methods for semantic segmentation of tomato plant parts

**DOI:** 10.3389/fpls.2023.1045545

**Published:** 2023-06-12

**Authors:** Bolai Xin, Ji Sun, Harm Bartholomeus, Gert Kootstra

**Affiliations:** ^1^Department of Plant Science, Wageningen University and Research, Wageningen, Netherlands; ^2^Laboratory of Geo-Information Science and Remote Sensing, Wageningen University and Research, Wageningen, Netherlands

**Keywords:** data augmentation, deep learning, point clouds, semantic segmentation, tomato plants

## Abstract

**Introduction:**

3D semantic segmentation of plant point clouds is an important step towards automatic plant phenotyping and crop modeling. Since traditional hand-designed methods for point-cloud processing face challenges in generalisation, current methods are based on deep neural network that learn to perform the 3D segmentation based on training data. However, these methods require a large annotated training set to perform well. Especially for 3D semantic segmentation, the collection of training data is highly labour intensitive and time consuming. Data augmentation has been shown to improve training on small training sets. However, it is unclear which data-augmentation methods are effective for 3D plant-part segmentation.

**Methods:**

In the proposed work, five novel data-augmentation methods (global cropping, brightness adjustment, leaf translation, leaf rotation, and leaf crossover) were proposed and compared to five existing methods (online down sampling, global jittering, global scaling, global rotation, and global translation). The methods were applied to PointNet++ for 3D semantic segmentation of the point clouds of three cultivars of tomato plants (Merlice, Brioso, and Gardener Delight). The point clouds were segmented into soil base, stick, stemwork, and other bio-structures.

**Results and disccusion:**

Among the data augmentation methods being proposed in this paper, leaf crossover indicated the most promising result which outperformed the existing ones. Leaf rotation (around Z axis), leaf translation, and cropping also performed well on the 3D tomato plant point clouds, which outperformed most of the existing work apart from global jittering. The proposed 3D data augmentation approaches significantly improve the overfitting caused by the limited training data. The improved plant-part segmentation further enables a more accurate reconstruction of the plant architecture.

## 1 Introduction

Plant scientists and plant breeders try to understand the relationship between the genotype, the environment, and the resulting phenotype. A key to understanding this relationship is to get large amounts of data. Genotypic data can nowadays be acquired easily using next-generation genotyping. The bottleneck, however, is in acquiring phenotypic data. In practice, plant phenotyping is often still performed by hand, resulting in low amount of data and suffering from the subjective interpretation of the human assessor. Some automatic high-throughput plant phenotyping systems are used, but these are often based on 2D images, which limit the accuracy of the estimation of geometric traits, such as internode diameter, and leaf area (Boogaard et al., 2020). With the rapid development of 3D sensors including 3D scanners, LiDARs and RGB-D cameras, more methods become available for 3D plant phenotyping, e.g., Johann et al. (2015); Golbach et al. (2016); Itakura and Hosoi (2018); Magistri et al. (2020); Boogaard et al. (2022). This paper has a focus on methods for 3D segmentation of plant parts as an important prerequisite to extract geometrical plant traits (Boogaard et al., 2021). Traditional methods for processing of 3D point clouds of plants generally achieves the segmentation through a set of manually designed algorithms. Itakura and Hosoi (2018), for example, projected the 3D point cloud to 2D planes, using a combination of watershed segmentation and morphological operators to get the leaf segments, which were then projected back to the 3D space and completed using region growing. In Lu et al. (2017) plant stems were segmented by calculating the local point density. Stem and leaves of tomato seedlings were segmented in Golbach et al. (2016) using a custom-made connected-component analysis method. More recently, Chebrolu et al. (2021) achieved leaf and stem segmentation using a support vector machine (SVM) based on fast point feature histograms (FPFH). However, these methods rely on human work to provide discriminative features for segmentation, a process which is sensitive to natural variations of plants even within a same cultivar, resulting in poor generalization (Heiwolt et al., 2021).

In recent years, 3D deep neural networks became available to extract discriminative features and perform segmentation in an end-to-end fashion. These methods learn to segment plant parts based on a set of training examples (van Dijk et al., 2020). Louëdec and Cielniak (2021), for example, used the position and surface normals of the 3D points as input, to learn to segment strawberries among canopies using an encoder-decoder convolutional neural network (CNN). Boogaard et al. (2021) presented a block-wise method for plant-part segmentation of full 3D point clouds based on PointNet++ (Qi et al., 2017b) showing enhanced performance when including spectral information alongside the spatial information. A stemwork segmentation methods was proposed in Ao et al. (2021) employing PointCNN (Li et al., 2018) followed by a refining process using random sampling consensus (RANSAC). Ma et al. (2021) presented a SPGNet-based (Landrieu and Simonovsky, 2018) segmentation method to segment the jujube tree stemwork into trunk and secondary branches.

These examples of the use of 3D deep neural networks indicate a more robust performance and better generalization compared to the traditional point-cloud processing algorithms. However, in order to perform well, these methods require a large number of labeled samples for training. As the labeling process is usually conducted manually, which is exceptionally tedious and time-consuming for 3D point clouds, there are typically only small labelled datasets available. This increases the risk of overfitting and generalization of the performance of the networks (Choi et al., 2021).

Data augmentation is a proven method to alleviate the problem of overfitting on small training sets. Data augmentation consists of small modifications of the original training data in order to enhance its diversity. In the domain of 3D point clouds, relevant data augmentation methods can be divided into global augmentation approaches and local augmentation approaches (Hahner et al., 2020). Global augmentation applies transformation on the whole point cloud. For example, Shi et al. (2020) and Chen et al. (2020) introduced random flipping, global scaling, global rotation, and global translation methods to enhance the training set, resulting in improved performance of 3D object detection in street scenes. In the agriculture domain, Ao et al. (2021) and Schunck et al. (2021) employed global rotation, global translation, and scaling operations to the original point clouds of maize and tomato, achieving the segmentation of stem and leaf parts. Li et al. (2022) proposed a down sampling-based augmentation method called 3D edge-preserving sampling (3DEPS) for the organ segmentation of tobacco, tomato, and sorghum point clouds. Local augmentation applies transformations to parts of the point cloud, typically to the parts that are relevant for the segmentation or detection task. Choi et al. (2021), for instance, proposed a combination of five types of local augmentation methods (dropout, swapping, mixing, down sampling, and noise adding), achieving good performance in 3D object detection in street scenes. To achieve the same goal, Hahner et al. (2020) introduced local translation, local rotation, and local scaling to enhance the point cloud data set. Although these local data-augmentation methods have shown their benefits in the domain of autonomous driving, they have not been used for 3D plant-part segmentation, possibly due to the complexity in plant architecture.

In this paper, we conducted a deep study on 3D data augmentation methods applied to the deep-learning-based plant part segmentation with insufficient training data. Five novel data augmentation methods for 3D semantic segmentation of plants were proposed, including two global augmentations (cropping and brightness adjustment) and three local augmentations (leaf translation, leaf rotation, and leaf crossover). Additionally, we also tested five commonly used augmentation methods (random down sampling, jittering, global scaling, global rotation, and global translation) with the same tomato plant dataset, and provided a comprehensive comparison and discussion of different data augmentation methods combining with their specific application scope. The experiments were conducted on three commonly used tomato plant cultivars (Merlice, Brioso, and Gardener Delight). We used PointNet++ (Qi et al., 2017b) as the deep neural network for semantic-segmentation method as this is currently one of the best performing networks for the segmentation of plant parts in particular (Turgut et al., 2022) and for point-cloud segmentation in general (Guo et al., 2021). Corresponding results and conclusions of the presented work are also valid for other network architectures.

The remaining of the article is organized as follows. The methodology together with the evaluation metrics are introduced in Section 2. Results and relevant discussions of the experiment are presented in Section 3. The proposed work is finally concluded in Section 4.

## 2 Materials and methods

Aiming to improve the generalization of plant-part segmentation, ten different augmentation methods were investigated, as described in Section 2.3. The methods were tested and evaluated on 3D point clouds collected of tomato plants, as presented in Section 2.1. Section 2.2 describes the deep neural network used in this work. Finally, the evaluation metrics are presented in Section 2.4.

### 2.1 Data acquisition

#### 2.1.1 Tomato plants

To train and test the proposed methods, 3D point clouds of 40 tomato plants were collected and carefully annotated. Three different cultivars were used: 17 Merlice plants, 19 Brioso, and 4 Gardener Delight. The plants were grown in a greenhouse environment. 19 of the plants were scanned in the second week of growth, and 21 plants were scanned in the third week. The 40 point clouds captured from these plants were split into 35 point clouds for training and 5 point clouds for testing.

#### 2.1.2 Imaging system - Maxi-Marvin

**Figure 1:**
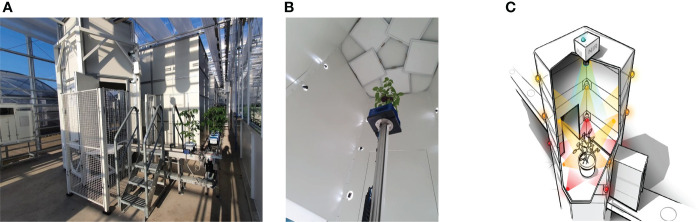
The imaging system - Maxi-Marvin - used to acquire point clouds of tomato plants in the proposed work. **(A)** Shows the global view of the system; **(B)** Shows the inner situation of the imaging black box when an exposure took place; **(C)** Shows a legend of the equipment setup inside the black box.

**Figure 2:**
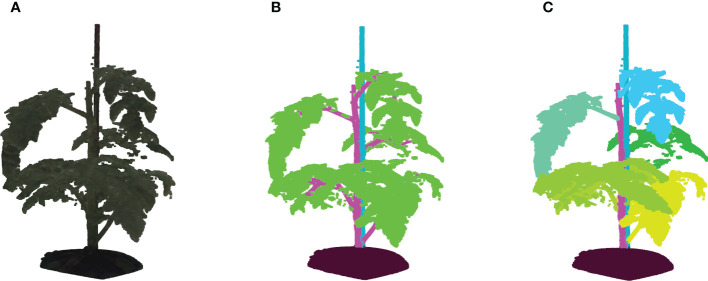
**(A)** Shows an example of a colored point cloud of Merlice, where the colour information being visualized is the median values of R, G, and B channels from 15 cameras, **(B)** Shows the corresponding semantic labels, with the classes soil base, stick, stemwork, and other bio-structures, marked by black, light blue, magenta, and green respectively, and **(C)** shows the leaf-instance labels.

The scanning of the tomato plants and the 3D point-cloud reconstruction was performed using a shape-from-silhouette method, similar to that used in Golbach et al. (2016). The setup is illustrated in **Figure 1**. Fifteen color cameras with a resolution of 1920×1080 were mounted in a cylindrical shape around the plant, on three different heights and five different angles. The fifteen cameras were synchronized to capture images at the exact same moment in time. In every camera image, the plant was segmented from the white background using color thresholds in the RGB color space, resulting in fifteen plant masks, each from a different viewpoint. A 3D voxel space in the center of the setup with a size of 40*cm*×40*cm*×70*cm* in length, width, and height respectively and a resolution of $1mm^{3}$ was used. For each voxel, it was determined if it was occupied or free by projecting it in the fifteen camera images based on the known and calibrated camera poses and intrinsic camera parameters. If a voxel projected in the plant mask for all camera images, it was considered occupied. Otherwise, if projected in the background of one or more camera images, it was deemed unoccupied. This shape-from-silhouette method resulted in a 3D voxel representation of the plant. By taking all the boundary voxels of the plant reconstruction, a 3D point cloud was obtained. Point-cloud data acquired with the system consists of 48 channels; three channels with positional information and 45 channels with RGB color information from each of the fifteen cameras. **Figure 2A** shows an example of a colored point cloud captured by the imaging setup. More details on the method can be found in Golbach et al. (2016). During the experiments, target plants were automatically transported to the imaging setup from the greenhouse using a conveyor-belt system. A single point cloud of a tomato plant contained 422,682 points on average.

#### 2.1.3 Point cloud labeling

Semantic labels and instance labels were given to all points in the point cloud during the manual labeling process. As shown in **Figure 2B**, semantic labeling divided points into four classes; soil base, stick, stemwork, and other bio-structures. The stemwork class consisted of the collection of the main stem, petioles^1^, rachis^2^, and petiolules^3^, while other bio-structures referred to all other plant organs including leaflets, flowers, and fruits.

Since the proposed work employed multiple local augmentation approaches, which required operations at the leaf level, instance labeling was also conducted to enable the localization of individual leaf instances. Different from semantic labeling, instance labeling gave a unique label to points of individual leaves. Here, a leaf instance consisted of the collection of leaflets, petioles, rachis, and petiolules belonging to that leaf. In this case, the class stemwork was reduced to a stem class, containing only the main stem. Thus, instance labeling consisted of the classes soil base, stick, stem, and all leaf instances as illustrated in **Figure 2C**. For some points, the class label could not be determined in the manual annotation process. These points were labeled as unclassified.

### 2.2 Network setup

PointNet++ (Qi et al., 2017b), one of the best performing deep neural networks for semantic segmentation of 3D point clouds (Guo et al., 2020) was used in this work to learn to segment the point cloud of a tomato plant into its different plant parts. The method is based on PointNet (Qi et al., 2017a), which learns to extract local point features and one global feature vector for the whole point cloud, which jointly enable the prediction of the class label for every point. To take the local context information into consideration, PointNet++ improves PointNet by adding a hierarchical composition and applying PointNet in three set-abstraction layers. Each abstraction layer uses a sampling and grouping operation to cluster points locally, followed by PointNet layers to extract features per cluster. This results in extracting local spatial features at that scale and down-sampling of the point cloud. The segmentation head uses feature propagation layers, which combine the local point features with the progressively more abstract cluster features. The resulting multi-scale feature description per point was then used to predict the semantic class labels. More details on PointNet++ can be found in (Qi et al., 2017b). To prevent overfitting on our small training set, we reduced PointNet++ to using two levels of abstraction instead of the three levels.

#### 2.2.1 Input point-cloud features

PointNet++ requires the network input to have a uniform size. By comprehensively considering the limitation of GPU memory and the minimum resolution needed for the plant-part segmentation task, we used *n*=50,000 points per point cloud in this paper. As the original point clouds of the plants contained nearly half a million of points, the point clouds needed to be down-sampled. More information on this down-sampling procedure is described in Section 2.3.

The input features per point are ${\text{p}}_{i}=\left[x_{i},y_{i},z_{i},n_{i}^{x},n_{i}^{y},n_{i}^{z},r_{i}^{1},g_{i}^{1},b_{i}^{1},\ldots ,r_{i}^{15},g_{i}^{15},b_{i}^{15}\right]$ , where $\left[x_{i},y_{i},z_{i}\right]$ is the 3D position of point *i*, [ $n_{i}^{x},n_{i}^{y},n_{i}^{z}$ ] is the 3D surface normal of the point and [ $r_{i}^{1},g_{i}^{1},b_{i}^{1},\ldots ,r_{i}^{15},g_{i}^{15},b_{i}^{15}$ ] is the RGB color information for the fifteen cameras. The point surface normals were included, as they likely contain relevant spatial information to separate the different classes. Color information was included because it mainly helps to distinguish bio-structures and non-bio-structures^4^. In order to normalize the magnitude of the input features, positional information of points were zero-centered and scaled in order to fit into a vertical (*z* axis) range of [0,1]. The surface normal had unit length, and the RGB color information was normalized into the range of [0,1].

Thus, a point cloud of a tomato plant is defined as $\text{P}=\{{\text{ p}}_{1,}{\text{p}}_{2},\ldots {\text{p}}_{\text{n}}$ } represented by a *n*×51 matrix.

#### 2.2.2 Hyper-parameter optimization

Necessary hyper parameters within the original PointNet++ include batch size, initial learning rate, and number of epoches. Due to the limitation of GPU memory, we employed a small batch size of five throughout the experiments. The number of epoches was selected as 400 for a clear observation of overfitting phenomenon with respect to each data augmentation method. The initial learning rate was selected with a 7-fold cross validation over the training set without using data augmentations, resulting in an optimal initial learning rate of 0.005.

### 2.3 Data-augmentation methods

The aim of this paper is to investigate the use of different data-augmentation methods to improve the generalization of plant-part segmentation when a small training set is used. In this section, several global and local data-augmentation methods are described. Based on Hahner et al. (2020), we selected five types of existing global augmentation methods (sampling, jittering, scaling, rotation, and translation). Apart from these, we proposed two new global augmentation methods (cropping and brightness adjustment) and three local augmentation methods (leaf translation, leaf rotation, and leaf crossover). These methods are described in detail in Sections 2.3.1 and 2.3.2.

#### 2.3.1 Global augmentation

Global augmentation employs slight alterations to the point features of all the points in a point cloud simultaneously. In this paper, we considered seven global augmentation methods, i.e. online down sampling, jittering, scaling, rotation, translation, cropping, and brightness adjustment.

##### 2.3.1.1 Online down sampling

**Figure 3:**
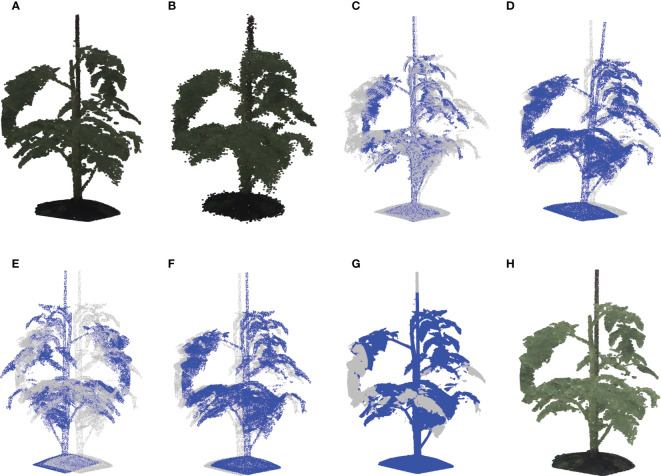
Examples of global augmentation operations. **(A)** Is the down sampling result based on the raw point cloud shown in **Figure 2A**. By taking the point cloud shown in **(A)** as the origin, **(B)** shows the jittering result with *σ*_j_ = 0.01, **(C)** is the scaled point cloud with respect to *y* axis employing a scaling rate of 0.75, **(D, E)** reveal the rotation results with respect to the *x* and *z* axes employing α=6° and γ=180° respectively, **(F)** shows the global translation result with δ=[0.02,0.05,0.005], and **(H)** shows the global brightness transform result with a brightness augmentation ratio of 2.0. By taking the raw point cloud shown in **Figure 2A** as the origin, **(G)** shows the cropping result with ϕ=0.1. The color information shown in **(A, B)**, and **(H)** is the median values of the R, G, and B channels over all cameras. In **(C–G)**, the original point clouds are marked by a semi-transparent color, while the transformed point clouds are marked by blue for a better comparison of the transformed point clouds with the origin.

The original point clouds contained nearly half a million of points and needed to be down-sampled to *n*=50,000 points. To this end, a random subset of *n* points was sampled from the original point cloud. As a data-augmentation method, this random down-sampling was used in an online mode, meaning that a new random sample was taken from the original point cloud after the acquisition of each training batch. This increases the variation in the training data, which should promote generalization. An example of a down-sampled point clouds is shown in **Figure 3A**.

When online down-sampling was not activated in the experiments, offline down-sampling was used, meaning that random sampling was applied only at the start of the training procedure as a pre-processing step, resulting in the same sampled subset used throughout training.

##### 2.3.1.2 Jittering

Jittering adds random fluctuations to the positions of individual points in the point cloud in order to simulate sensor noise and to create additional variation. The position of a point was changed by


(1) $$\left[x_{i}^{'},y_{i}^{'},z_{i}^{'}\right]=\left[x_{i},y_{i},z_{i}\right]+\left[\varepsilon_{i}^{x},\varepsilon_{i}^{y},\varepsilon_{i}^{z}\right]$$

where the added noise is drawn from a Gaussian distribution, ϵ_i_ ∼ *N(0,σ_j_)* for every point separately. Note that the position data was normalized into a z-coordinate range of [0,1], the noise level used here is proportional to the original height of a specific plant. During the experiments, ten different values for *σ_j_* were evaluated, 0.005, 0.01, 0.02, 0.03, 0.04, 0.05, 0.06, 0.07, 0.08, 0.09, and 0.11 respectively. **Figure 3B** shows an example of jittering with *σ_j_* = 0.01.

##### 2.3.1.3 Scaling

The scaling operation enlarges or shrinks the original point cloud with a certain factor to introduce variation in the plant size. As the position data was normalized with respect to the height of the plant, scaling was only applied to the x- and y-coordinates:


(2) $$\left[x_{i}^{'},y_{i}^{'},z_{i}^{'}\right]=\left[\eta_{x}\cdot x_{i},\;\eta_{y}\cdot y_{i},z_{i}\right]$$

For every point cloud in every training batch, the scaling factors were randomly drawn from a uniform distribution: η∼*U*(*a*,*b*), with *a* and *b* being the lower and upper bound of the uniform distribution. During the experiments, different ranges were evaluated using *U*(0.9,1.1), *U*(0.8,1.2), *U*(0.7,1.3), *U*(0.6,1.4), and *U*(0.5,1.5). **Figure 3C** shows an example with *η_y_* = 0.75.

###### 2.3.1.3.1 Rotation

**Figure 4:**
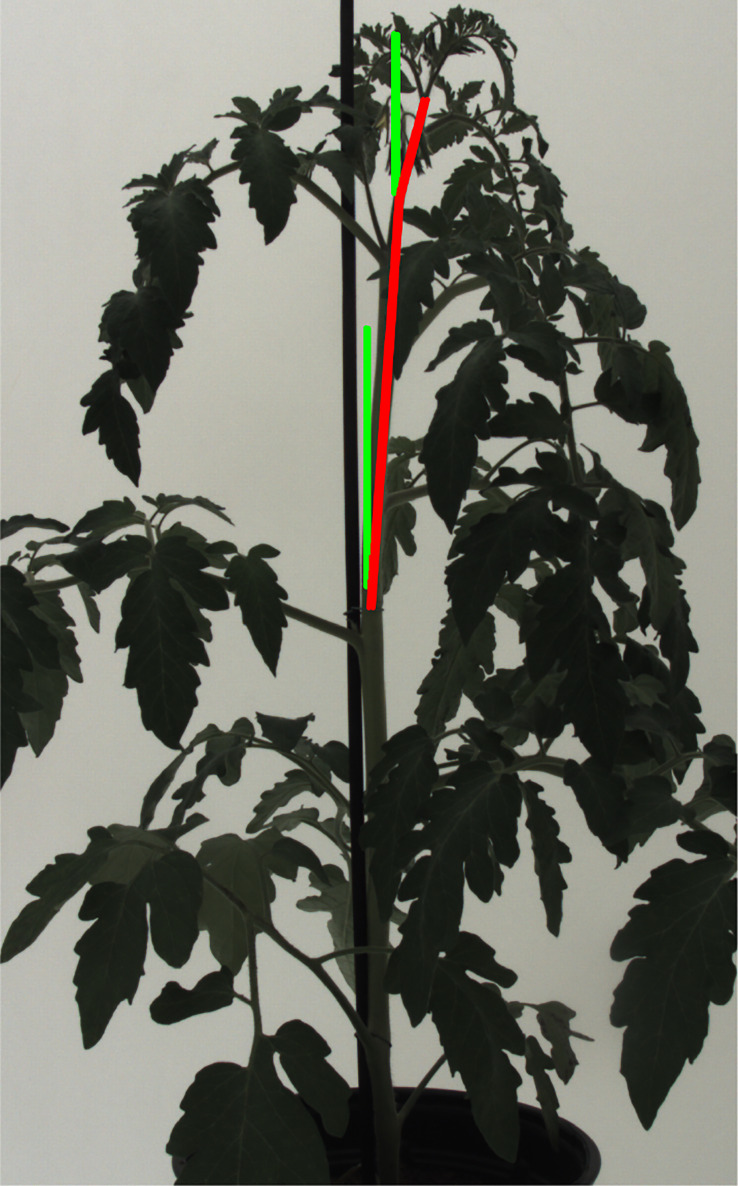
The inclination of stem internodes at the top part of a plant. Here, the growing trend of the top part is marked with red lines while the perpendicular orientation is marked by green lines for a comparison.


The global rotation operation rotates the original point cloud with random angles for each of the three axes. A rotation around the z-axis, $R_{z}(\gamma )$ , corresponds to variation in the rotation of the plant in the imaging system. Rotations around the x- and y-axes, ${\text{R}}_{\text{x}}(\alpha )$ and ${\text{R}}_{\text{y}}(\beta )$ , correspond to a change in the inclination of the stem (**Figure 4**). The rotation changes the position of all the points in the point cloud according to:

(3) $${\left[x_{i}^{'},y_{i}^{'},z_{i}^{'}\right]}^{T}=R\cdot {\left[x_{i},y_{i},z_{i}\right]}^{T}$$

where the rotation matrix is $R={\text{R}}_{\text{z}}\left(\gamma \right)R_{y}\left(\beta \right)R_{x}\left(\alpha \right)$ . The rotation around the z-axis was drawn from a uniform distribution, $\gamma ∼U\left(0^{{}^{\circ}},360^{{}^{\circ}}\right)$ , and the rotations around the x- and y-axes are drawn from a Gaussian distribution, $\alpha ,\beta ∼N\left(0,\sigma_{r}\right)$ . During the experiments, different values for α and β were evaluated: 1°, 2°, 2.25°, 3°, and 4°. **Figures 3D, E** show two examples of global rotation around the *x* and *z* axes.

###### 2.3.1.3.2 Translation

The translation operation shifts the complete point cloud with a random offset:


(4) $$\left[x_{i}^{'},y_{i}^{'},z_{i}^{'}\right]=\left[x_{i},y_{i},z_{i}\right]+\left[\delta^{x},\delta^{y},\delta^{z}\right]$$

where the random offset is drawn from a uniform distribution, δ∼*U*(*a*,*b*), with lower and upper bounds *a* and *b*. This introduces some variation in the position of the plant in the point clouds, with the aim to train the network to be robust to changes in position. Note that the position data was normalized to have *z* in the range of [0,1], hence the offset is proportional to the height of the plant. During the experiments, the use of different ranges of the uniform distribution were evaluated, for *U*(−0.03,0.03), *U*(−0.05,0.05), *U*(−0.10,0.10), *U*(−0.15,0.15), and *U*(−0.40,0.40). An example with δ=[0.02,0.05,0.005] can be shown in **Figure 3F**.

###### 2.3.1.3.3 Cropping

The imaging system (Section 2.1.2) has a limited working range, which is sometimes smaller than the plant. This results in parts of the plant being cut-off at the boundaries of the working space at the left, right, front, back, and top. To train the model to be more robust to such incomplete point clouds, a random crop was applied as data-augmentation method. The size of the crop was parameterized with the factor *ϕ*, drawn from a uniform distribution, *ϕ*∼*U*(*a*,*b*). A point, *i*, remained in the point cloud if:


(5) $$\begin{array}{l} x_{i}\in \left[\left(1\;-\;\phi \right)\cdot \text{min}\left(x\right),\left(1\;-\;\phi \right)\cdot \text{max}\left(x\right)\right]\wedge \\ y_{i}\in \left[\left(1\;-\;\phi \right)\cdot \text{min}\left(y\right),\left(1\;-\;\phi \right)\cdot \text{max}\left(y\right)\right]\wedge \\ z_{i}\in \left[0,\left(1\;-\;\phi \right)\cdot \text{max}\left(z\right)\right] \end{array}$$

where the operators min(…) and max(…) give the minimum and maximum value along the respective axis. Mind that the x- and y-coordinates were zero-centered and the z-coordinates were in the range [0,1] as a result of the normalization process. As the cropping operation results in fewer points in the point cloud, the online down sampling process Section 2.3.1.1 was applied after cropping, to ensure that the resulting point cloud after data augmentation contains *n*=50,000 points. During the experiments, the following ranges of the uniform distribution were evaluated: *U*(0,0.05), *U*(0,0.10), *U*(0,0.15), and *U*(0,0.20). **Figure 3G** shows an example of a cropped point cloud using *ϕ* =0.1.

###### 2.3.1.3.4 Brightness adjustment

This data-augmentation methods introduces variation in the brightness of the colors in the point clouds to become more robust to changes in illumination. The brightness is altered with a random factor drawn from a uniform distribution, η∼*U*(*a*,*b*). All color channels are then altered by multiplying with this factor:


(6) $$\left[{r_{i}^{'}}^{1},{g_{i}^{'}}^{1},{b_{i}^{'}}^{1},\ldots ,{r_{i}^{'}}^{15},{g_{i}^{'}}^{15},{b_{i}^{'}}^{15}\right]=\left[\eta \cdot r_{i}^{1},\eta \cdot g_{i}^{1},\eta \cdot b_{i}^{1},\ldots ,\eta \cdot r_{i}^{15},\eta \cdot g_{i}^{15},\eta \cdot b_{i}^{15}\right]$$

In the experiments, different ranges of the uniform distribution were evaluated for *U*(0.4,3.5), *U*(0.5,3.0), *U*(0.6,2.5), and *U*(0.7,2.0). **Figure 3H** shows an example of the modified brightness for η=2.0.

##### 2.3.1.4 Local augmentation

Different from the global augmentation methods, the local augmentation methods alter a subset of the point cloud. In the proposed work, three types of local augmentation approaches have been proposed that operate on the leaf level: leaf translation, leaf rotation, and leaf crossover. This enhances the diversity of the plant architectures in the training set.

**Figure 5:**
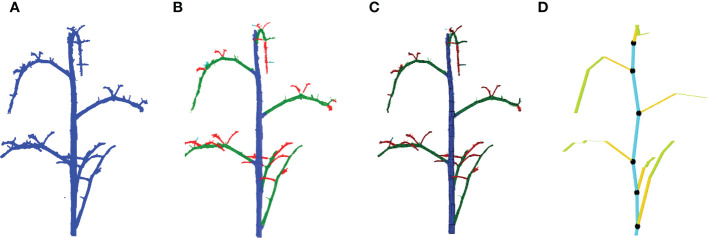
Leaf base point detection achieved with TreeQSM. **(A)** Reveals a clean stemwork point cloud of tomato plant, which is taken as the input of TreeQSM; **(B)** is the result of hierarchical analysis; **(C)** is the reconstruction result generated by TreeQSM pipeline; **(D)** reveals the positions of leaf base points over the stemwork reconstruction model. Here, the first internodes of individual second order branches (petioles) are marked by orange. The black dots refer to the base points of leaves, which is defined as the connection part between leaves and their parent stem internodes.

As a basis of the local augmentation methods, we need to know the position and the orientation of every leaf in the point clouds in the training set. A method was developed to automatically calculated these features based on Tree Quantitative Structural Modeling (TreeQSM) (Raumonen et al., 2013). TreeQSM is a method to model the 3D architecture of a tree or plant by fitting a set of connected cylinders to point-cloud data of the stemwork (main stem and all side branches). TreeQSM requires a clean point cloud of the stemwork as input (see **Figure 5A**), which we can obtain from the annotated point clouds within the training set. TreeQSM then fits a series of connected cylinders to the points in order to get an hierarchical reconstruction of the architecture with the main stem and second- and higher-order branches. An example is given in **Figure 5B**, where points marked by blue refer to the main stem, points marked by green refer to second-order branches (petioles and rachis of individual leaves), while points marked by red stand for third-order branches (petiolules of individual leaves). The cylinder-based 3D reconstruction can be observed in **Figure 5C**.

To obtain the position of the leaf, the method determined the base points of the leaves by taking the starting point of the first-order branches reconstructed by TreeQSM, as illustrated in **Figure 5D** by black dots.

**Figure 6:**
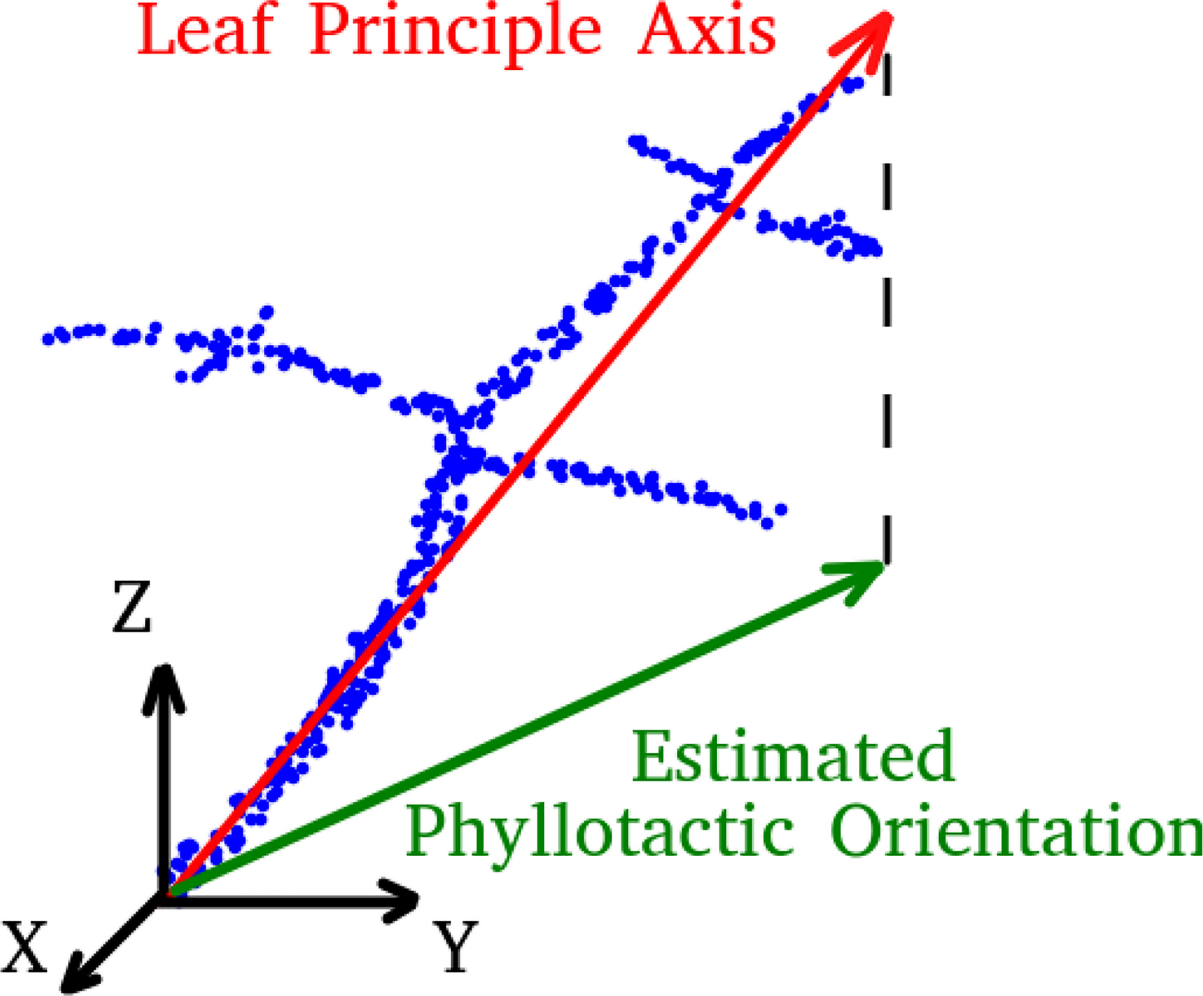
A legend of leaf principle axis and estimated phyllotactic orientation. Blue dots refer to the point cloud of leaf stemwork.

The orientation of each leaf is determined by the first principle component calculated from a principle component analysis (PCA) of all points belonging to the stemwork of that leaf, as illustrated in **Figure 6**. The resulting leaf principle axis is a 3D vector representing the orientation of the leaf. The phyllotactic orientation of the leaf is then approached by taking the projection of this 3D vector onto the *xoy*-plane. The angle between the phyllotactic orientation vectors of two adjacent leaves is defined as the phyllotactic angle.

###### 2.3.1.4.1 Leaf translation

**Figure 7:**
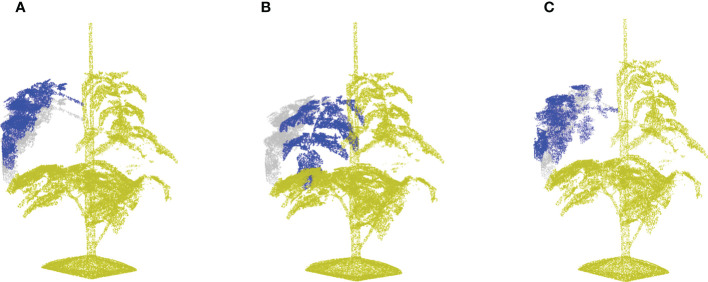
A demonstration of local data augmentation by taking the point cloud in **Figure 3A** as origin. **(A)** Shows the leaf translation result of the leaf at rank five with a relative translation distance of 0.1. **(B)** Shows a leaf rotation around the vertical axis with γ =60°, and **(C)** shows the leaf rotation around the leaf principle axis with θ = 60^◦^.

This local augmentation method applies a random translation along the z-axis to the position of individual leafs, with a different offset for every leaf. In this manner, leaf translation is able to simulate the potential fluctuations of leaf positions on the stem, from which enhance the diversity of stem internode lengths. The set of points, ${\text{L}}_{\text{j}}$ , belonging to a specific leaf *j* is selected based on the annotated leaf-instance labels (see Section 2.1.3), where ${\text{L}}_{\text{j}}\subset \text{P}$ . Then, for every point ${\text{I}}_{\text{i}}\in {\text{L}}_{\text{j}}$ , the position is updated by


(7) $$\left[x_{i}^{'},y_{i}^{'},z_{i}^{'}\right]=\left[x_{i},y_{i},z_{i}\right]+\left[0,0,\psi_{z}\right]$$

where $\psi_{z}$ is drawn from a Gaussian distribution, $\psi_{z}∼N(0,\sigma_{l})$ . During the experiment, evaluations were performed for different values of $\sigma_{l}$ : 0.10, 0.15, 0.20, 0.25, 0.30, 0.40, 0.50, 0.60, and 0.70. Note that the position data was normalized to have *z* in the range of [0,1], hence the offset is proportional to the height of the plant. An example of leaf translation operation is shown in **Figure 7A**. Here, the original position of the leaf is marked as a semi-transparent color, the leaf after translation is marked as blue, and all parts that remain the same are marked as yellow.

###### 2.3.1.4.2 Leaf rotation

The leaf-rotation method applies two types of random rotations for every leaf on the plant. One rotation is around the vertical axis to change the phyllotactic angle of the leaf. The other one is a rotation around the leaf principle axis, which aims to simulate the situation that a leaf may slightly alter its orientation according to the space utilization and the sunshine direction. For both rotations, necessary translation operations are applied to the target leaf point cloud ${\text{L}}_{\text{j}}$ in order to assure the rotations to be conducted around the base point of the target leaf.

For the change in phyllotactic angle, the points are rotated around the vertical axis (z-axis) over a randomly picked angle, γ:


(8) $${\left[x_{i}^{'},y_{i}^{'},z_{i}^{'}\right]}^{T}={\text{R}}_{z}\left(\gamma \right)\cdot {\left[x_{i},y_{i},z_{i}\right]}^{T}$$

where ${\text{R}}_{z}\left(\gamma \right)$ is a 3×3 rotation matrix corresponding to the rotation around the z-axis with an angle of γ. The random angle was drawn from a uniform distribution, γ∼*U*(−*a*,*a*). During the experiments, different values of *a* were evaluated: a=30°, a=90°, a=135°, a=180°. An example of leaf rotation of with respect to *z* axis can be seen in **Figure 7B**.

The leaf rotation with respect to the principle axis is achieved by


(9) $${\left[x_{i}^{'},y_{i}^{'},z_{i}^{'}\right]}^{T}={\text{R}}_{\text{v}}\left(\text{v},\;\theta \right)\;\cdot \;{\left[x_{i},y_{i},z_{i}\right]}^{T}$$

where ***R*_v_** (***v***,*θ*) is a rotation matrix corresponding to the rotation angle θ around the axis **v** ^5^, which here was the leaf principle axis. The random angle was drawn from a uniform distribution, θ∼*U*(−*b*,*b*). During the experiments, different values of *b* were evaluated: *b*=30°, *b*=40°, *b*=50°, *b*=60°, and *b*=70°. An example of leaf rotation around the leaf principle axis can be seen in **Figure 7C**.

###### 2.3.1.4.3 Leaf crossover

**Figure 8:**
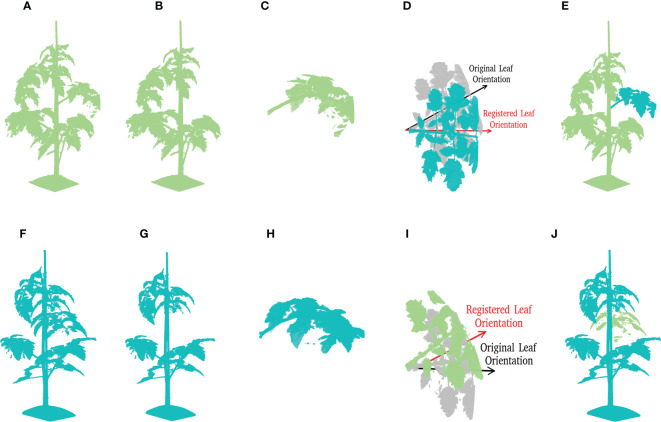
A demonstration of leaf crossover process. Suppose that there are two tomato plant point clouds as shown in **(A, F)**, this example is going to reveal the crossover process of leaves at rank four in details. For a better visualisation, plant parts coming from **(A, F)** are marked by pale green and cyan respectively through out the process. **(B, G)** Reveal the incomplete point clouds after leaves selected for further crossover operation are removed. **(C, H)** show the cut-off leaf candidates. To maintain the original phyllotactic angle of leaves, the leaf orientation is calibrated as shown in **(D, I)**, where the original orientation is marked by a semi-transparent colour. The final results after crossover are shown in **(E, J)** for respective plants.

Leaf crossover aims to exchange leaf instances among different plant samples in order to enhance the diversity of plant appearances. To create meaningful transformations, leaves were only crossed-over within one cultivar. **Figure 8** shows the entire crossover process of two plant candidates. The leaf crossover operation contained the following steps:

Step 1: Selection of all plants in the training set of cultivar A, creating the set ${\mathbb{P}}_{0}^{A}=\left\{P_{1}^{A},\ldots P_{m}^{A}\right\}$ .

Step 2: Random selection of a morphological rank *r*, which is based on the order of the leaf on the stem, from bottom (rank 1) to top (rank *n_leaf_*). Knowing that the size and shape of the leaf relate to the leaf age, we only swapped leaves with the same rank in the crossover process in order to prevent unrealistic appearance.

Step 3: Cutting-off the leaf with rank *r* of all plants in ${\mathbb{P}}_{0}^{A}$ . Based on the annotated leaf instances, all points of the leaf with rank *r* on plant *i* are selected to form the leaf point cloud, $L_{i}^{A,r}$ . This leaf is then cut-off to form a new point cloud of the plant without the selected leaf: ${\hat{P}}_{i}^{A}=P_{i}^{A}⦵L_{i}^{A,r}$ . **Figures 8B, G** show two examples of the point clouds without the selected leaves, and **Figures 8C, H** show the leaf point clouds being removed.

Step 4: Crossover of the leaves. Every cut-off leaf of plant *i* is added to a randomly selected other plant *j*: ${\bar{P}}_{j}^{A}={\hat{P}}_{j}^{A}⊕L_{i}^{A,r}\cdot T_{i\to j}$ , where *i*≠*j* and ${\text{T}}_{i\to j}$ is the transformation matrix to align leaf *i* with the position and phyllotactic angle of the original leaf *j*. Note that this matrix multiplication applies only to the position data of the point cloud. **Figures 8D, I** show the registration of the selected leaf to match the orientation of the original leaf. And **Figures 8E, J** contain the new point clouds resulting from the crossover process.

Step 5: Steps 2-4 are repeated $N_{C}$ times, that is *N_C_* leaves are crossed-over per plant.

Step 6: Steps 1-5 are repeated for all cultivars.

Due to the fact that the leaf crossover operation changed the number of points within the point cloud, the down sampling of the resulting point clouds was performed after the operation to maintain a fixed size of the point cloud. The numbers of leaves $N_{C}$ being exchanged per plant during the crossover process was evaluated for 1, 2, 4, 6, and 8 in the experiment.

#### 2.3.2 Evaluation

In the experiments, the network was trained and tested on a work station with an Intel Xeon E-2276M (2.8GHz) CPU and a NVIDIA Quadro RTX 5000 Max-Q (16GB) GPU.

The point clouds in the test set were down-sampled in an offline mode to uniform number of points (*n*=50,000). To evaluate the improvement of individual data-augmentation methods on the performance of semantic segmentation, the F1-score was used. This is calculated as the harmonic mean of the precision, $\frac{TP}{TP+FP}$ , and recall $\frac{TP}{TP+FN}$ , which are based on the number of true positives (TP), false positives (FP), and false negatives (FN) per semantic class(soil, stick, stemwork, and other bio-structure). The F1-score ranges from 0 (bad) to 1 (perfect). The F1-score was calculated per semantic class and averaged over the classes.

Considering that the randomness in the network initialization may give rise to certain fluctuation in the segmentation *F*1 score, totally five runs of training and testing were conducted for each data augmentation method. The final performance of the data augmentation method was described by the average performance over the five runs.

As a baseline, the PointNet++ model was trained without the use of data augmentation and compared to training using the presented augmentation methods, one at a time. For every data-augmentation method, different settings for the associated parameters were tested. In the results section, we selected the optimal parameter setting using the F1-score for stemwork. The reason for this was twofold: (1) the stemwork is the most challenging class and (2) the stemwork is very important to extract phenotypic traits like internode length and leaf orientation.

## 3 Results and discussion

The baseline result revealed average *F*1 scores of 0.97, 0.76, 0.47, and 0.94 for classes of soil base, stick, stemwork, and other bio-structures respectively. The baseline result sets the basis for the following comparison and evaluation. The content of this section is organized as follows. The results of the individual data augmentation methods are presented and discussed together with the optimal parameter selections in Section 3.1. In Section 3.2, we provide general discussions regarding the data augmentation methods being used in this paper.

### 3.1 Specific augmentation methods

In this sub-section, the segmentation performance achieved with the different data augmentation methods is presented together with corresponding optimal parameter selections. The optimal parameter selection was based on the metric of average stemwork *F*1 score. This is mainly because the segmentation of stemwork is much more difficult than that of other classes due to the low separability. Further discussion on this point is provided in Section 3.2. For the convenience of comparison, an integrated table is presented by combining the stemwork *F*1 scores and class-average *F*1 scores of all the data augmentation methods and parameter selections (**Table 1**).

**Table 1:** Performance of data augmentation methods with different parameter selections.

Augmentation Methods	Parameters	Stemwork F1 Scores	Class-ave F1 Scores	Augmentation Methods	Parameters	Stemwork *F*1 Scores	Class-ave *F*1 Scores
Baseline	- -	0.47	0.79	Cropping	*U* (0,0.10)	0.57 (+0.10)	0.83 (+0.04)
Down Sampling	- -*	0.58 (+0.11)	0.83 (+0.04)		*U* (0,0.15)*	0.59 (+0.12)	0.84 (+0.05)
Jittering	*σ_j_* = 0.005	0.47 (+0.00)	0.79 (+0.00)		*U* (0,0.20)	0.57 (+0.10)	0.83 (+0.04)
	*σ_j_* = 0.01	0.48 (+0.01)	0.79 (+0.00)	Brightness Adjustment	*U* (0.7.2.0)	0.52 (+0.05)	0.81 (+0.02)
	*σ_j_* = 0.02	0.52 (+0.05)	0.81 (+0.02)		*U* (0.6,2.5)	0.55 (+0.08)	0.82 (+0.03)
	*σ_j_* = 0.03	0.56 (+0.09)	0.83 (+0.04)		*U* (0.5.3.0)*	0.56 (+0.09)	0.81 (+0.02)
	*σ_j_* = 0.04	0.61 (+0.14)	0.84 (+0.05)		*U* (0.4,3.5)	0.55 (+0.08)	0.81 (+0.02)
	*σ_j_* = 0.05	0.60 (+0.13)	0.84 (+0.05)	Leaf Translation	*σ_l_* = 0.10	0.52 (+0.05)	0.80 (+0.01)
	*σ_j_* = 0.06	0.61 (+0.14)	0.84 (+0.05)		*σ_l_* = 0.15	0.55 (+0.08)	0.82 (+0.03)
	*σ_j_* = 0.07	0.60 (+0.13)	0.84 (+0.05)		*σ_l_* = 0.20	0.57 (+0.10)	0.82 (+0.03)
	*σ_j_* = 0.08*	0.63 (+0.16)	0.85 (+0.06)		*σ_l_* = 0.25	0.56 (+0.09)	0.81 (+0.02)
	*σ_j_* = 0.09	0.63 (+0.16)	0.84 (+0.05)		*σ_l_* = 0.30	0.55 (+0.08)	0.82 (+0.03)
	*σ_j_* = 0.11	0.63 (+0.16)	0.85 (+0.06)		*σ_l_* = 0.40	0.59 (+0.12)	0.83 (+0.04)
Scaling	*U* (0.9.1.1)	0.46 (-0.01)	0.78 (-0.01)		*σ_l_* = 0.50	0.60 (+0.13)	0.83 (+0.04)
	*U* (0.8,1.2)	0.49 (+0.02)	0.80 (+0.01)		*σ_l_* = 0.60	0.57 (+0.10)	0.81 (+0.02)
	*U* (0.7,1.3)	0.50 (+0.03)	0.80 (+0.01)		*σ_l_* = 0.70*	0.60 (+0.13)	0.83 (+0.04)
	*U* (0.6,1.4)	0.48 (+0.01)	0.80 (+0.01)	Leaf Rotation (*z*)	*U* (-30°,30°)	0.56 (+0.09)	0.82 (+0.03)
	*U* (0.5.1.5)*	0.51 (+0.04)	0.81 (+0.02)		*U* (-90°,90°)	0.59 (+0.12)	0.83 (+0.04)
Rotation (*z*)	*U* (0,360°)*	0.58 (+0.11)	0.83 (+0.04)		*U* (-135°,135)*	0.62 (+0.15)	0.84 (+0.05)
Rotation (*x* or *y*)	*σ_r_* = 1° *	0.50 (+0.03)	0.80 (+0.01)		*U* (-180°,180°)	0.60 (+0.13)	0.84 (+0.05)
	*σ_r_* = 2°	0.47 (+0.00)	0.79 (+0.00)	Leaf Rotation (PCA)	*U* (-30°,30°)	0.52 (+0.05)	0.81 (+0.02)
	*σ_r_* = 2.25°	0.46 (-0.01)	0.79 (+0.00)		*U* (-40°,40°)	0.53 (+0.06)	0.81 (+0.02)
	*σ_r_* = 3°	0.49 (+0.02)	0.80 (+0.01)		*U* (-50°,50°)*	0.54 (+0.07)	0.82 (+0.03)
	*σ_r_* = 4°	0.49 (+0.02)	0.80 (+0.01)		*U* (-60°,60°)	0.52 (+0.05)	0.81 (+0.02)
Translation	*U* (-0.03,0.03)	0.46 (-0.01)	0.79 (+0.00)		*U* (-70°,70°)	0.53 (+0.06)	0.82 (+0.03)
	*U* (-0.05.0.05)*	0.48 (+0.01)	0.79 (+0.00)	Leaf Crossover	*N_c_* = 1	0.61 (+0.14)	0.84 (+0.05)
	*U* (-0.10.0.10)	0.47 (+0.00)	0.79 (+0.00)		*N_c_* = 2	0.61 (+0.14)	0.84 (+0.05)
	*U* (-0.15,0.15)	0.46 (-0.01)	0.79 (+0.00)		*N_c_* = 4*	**0.65 (+0.18)**	**0.86 (+0.07)**
	*U* (-0.40,0.40)	0.45 (-0.02)	0.79 (+0.00)		*N_c_* = 6	0.64 (+0.17)	0.85 (+0.06)
Cropping	*U* (0,0.05)	0.58 (+0.11)	0.83 (+0.04)		*N_c_* = 8	0.62 (+0.15)	0.85 (+0.06)

For columns "Stemwork *F*1 Scores" and "Class-ave *F*1 Scores", values within the bracket refer to the improvement over the baseline result. The optimal parameters for individual augmentations are marked by *. The overall best performance is emphasised with bold font.

#### 3.1.1 Online down sampling

The segmentation with online down sampling data augmentation resulted in an average stemwork *F*1 score of 0.58, which was 0.11 higher than the baseline result (**Table 1**). Knowing that the test point clouds were also down sampled in an offline mode (Section 2.4), the online down sampling augmentation properly simulated the diversity and randomness of the test set, which makes the improvement easy to understand. Online down sampling did not include any parameters to be selected. Therefore, no information on optimal parameter selection is provided. As mentioned in Section 2.3.1.1, we used a uniform down sampling strategy to augment the dataset diversity in the experiment. However, the point clouds obtained from imaging systems usually have different densities depending on the distances between the plant parts and the imaging equipment. When that is the case, it is also possible to apply a non-uniform down sampling strategy to enhance the diversity of local areas, which lays a foundation for further improvements.

#### 3.1.2 Global jittering

**Figure 9:**
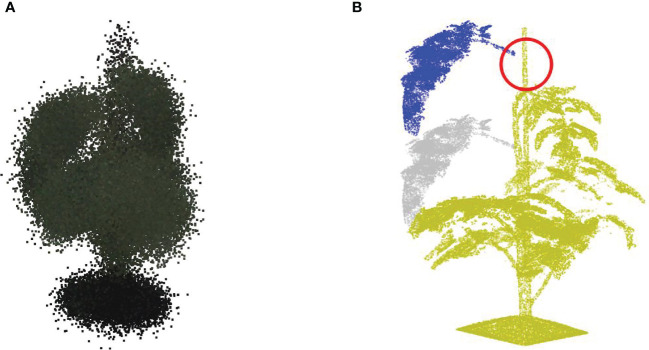
Potential deformation caused by the data augmentation operation with extreme parameter selections. **(A)** Reveals the jittering results with σ*_j_* =0.05 (32.8mm specifically for this plant); **(B)** Reveals the leaf translation result with a proportional translation distance of 0.5 (328 mm specifically for this plant).

The performance of jittering augmentation revealed a gradually increasing performance as the $\sigma_{j}$ value becomes larger, as shown in **Table 1**. This is because a larger $\sigma_{j}$ value enables larger variations of each training point cloud, which was more likely to generalize the actual diversity of the test set. However, as the value of $\sigma_{j}$ becoming larger, the fluctuation of point positions was becoming larger as well, which gave rise to a serious deformation of the original point cloud. For example, **Figure 9A** reveals an example of jittering operation with $\sigma_{j}=0.05$ , where most of the organs are no longer distinguishable purely based on the position of points. In this case, the positional information of points was no longer a representative feature when conducting the identification of certain classes, for example, the segmentation between stemwork and stick. Consequently, the network focused on other information types, for instance, color information, instead of the positional information in order to make a correct identification. In this circumstance, the fluctuation of positional information controlled by jittering $\sigma_{j}$ was not able to influence the segmentation performance anymore. Therefore, the segmentation performance was no longer sensitive towards $\sigma_{j}$ changes when $\sigma_{j}>0.04$ .

#### 3.1.3 Global scaling

According to **Table 1**, global scaling augmentation did not show a remarkable improvement compared to the baseline result, and different scaling ranges did not lead to significant variations in the stemwork segmentation performance. Global scaling augmentation aims to simulate the diversity of plant width, but the plant widths of the test point clouds after point cloud normalization did not show a large variation. As a result, the global scaling operation was not able to properly generalize the diversity of the test set.

#### 3.1.4 Global rotation

Global rotation augmentation with respect to the *Z* axis resulted in an average stemwork segmentation *F*1 score of 0.58, which was 0.11 higher than the baseline result. This is a further confirmation on the hypothesis that point cloud orientation was one of the main factors contributing to the diversity of the dataset.

Global rotation augmentation with respect to the *X* or *Y* axis did not indicate a significant improvement compared to the baseline result according to **Table 1**. The purpose of employing the global rotation augmentation with respect to *X* or *Y* axis is to simulate the stem inclination, but the result shows that this was not a good method to simulate the stem inclination variation. As shown in **Figure 4**, only the top part of the stem shows inclination in most occasions. However, the rotation along the *X* or *Y* axis made the complete plant point cloud to be leaning, which was not matching the real conditions and drove the enhancement of the training data diversity into a wrong direction. Another reason why global rotation around the *X* or *Y* axis did not improve the model is that there was only one point cloud within the test set that had a significant inclination of the stem. Therefore, the increase of training data diversity with respect to stem inclination could not generalize the diversity in the test set, which obstructed the augmentation method to work as intended.

#### 3.1.5 Global translation

According to **Table 1**, global translation augmentation did not indicated significant increment on the segmentation performance. This suggested that the positional variations of point clouds actually was not the main issue contributing to the dataset diversity.

#### 3.1.6 Cropping

As for the global cropping augmentation, the segmentation performance demonstrated a large improvement comparing to the baseline result, as shown in **Table 1**. However, it was insufficient to conclude that the cropping augmentation itself contributed to the improvement because the cropping operation was always followed by an online down sampling process. Knowing that the online down sampling improved the stemwork segmentation *F*1 score by 0.11, the cropping operation actually did not provide an outstanding improvement over that. Moreover, there was not a significant difference in segmentation performance with different cropping ratios, which suggests that the limitation of imaging scope was not a major sources of the dataset diversity.

#### 3.1.7 Brightness adjustment

Brightness adjustment achieved an obvious improvement compared to the baseline result, as shown in **Table 1**. There were two potential factors resulting in the improvement. Firstly, as discussed in Section 2.3.1.7, there existed certain variations in brightness during the imaging process, which increased the diversity in the dataset. With brightness adjustment the diversity of the training data was properly enriched, which improved the stemwork segmentation. Generally, the color information of point clouds within the dataset show a low brightness due to the exposure settings. A brightness transform, in particular a brightness enhancement operation, largely contributed to the separability of color features for respective classes. This also explains why the augmentation performance was better using a wider brightness enhancement range. There are several feasible approaches for brightness adjustment being proposed in literature, such as gray-shade color constancy adjustment (Finlayson and Trezzi, 2004) and gray-world color enhancement (Kwok et al., 2011). Only one type of brightness adjustment method was actually employed in this paper, but testing other types of brightness adjustment algorithms is meaningful in future work to explore the optimal solution. Besides, giving that the color information of a point cloud was provided by the images from 15 cameras (Section 2.1.2), the proposed brightness adjustment augmentation altered the brightness of these 15 camera images with a same strategy. Further studies can also investigate the feasibility of adjusting the brightness of each camera image with different strategies.

#### 3.1.8 Leaf translation

With an increase of leaf translation $\sigma_{l}$ , the stemwork segmentation performance demonstrated a gradually increasing trend, as shown in **Table 1**. This suggests that the lengths of stem internodes was one of the main source of the dataset diversity. However, the stemwork segmentation *F*1 score indicated a convergence after $\sigma_{l}\geq 0.4$ . Similar phenomenon was also observed with the jittering, as described in Section 3.1.2. When the $\sigma_{l}$ value increases, the deformation of the augmented point clouds increased as well, until passing an acceptable deformation level. For example, certain leaves might not be properly connected with the main stem after the leaf translation with a large distance, as shown in **Figure 9B**. Once the deformation was large enough and the positional information was no longer the most representative information to identify the stemwork, the network turned to other types of features (for example, color information) instead. Under this circumstance, the positional information did not have a significant influence on the segmentation performance anymore. The reason why the stemwork *F*1 score was no longer sensitive for the leaf translation $\sigma_{l}$ can be explained. In this paper, the translation distance of a leaf candidate was assumed to be independent from the positions of other leaf candidates. This easily gave rise to certain abnormal lengths of stem internodes (extremely long or short stem internodes), which was the main reason for the unexpected deformation. To improve this, certain constraints based on the statistic analysis on stem internode lengths can be applied to the selection of leaf translation distances to provide a better generalization of the real circumstance.

#### 3.1.9 Leaf rotation

In terms of the leaf rotation augmentation with respect to *z* axis, an increasing trend was observed for the stemwork segmentation *F*1 score as the rotation range became larger (**Table 1**). According to Section 2.3.2.2, the rotation angles for individual leaves was supposed to respect a uniform distribution. This meant that the phyllotactic orientation of a leaf was assumed to be random and independent from the orientations of other leaves. In this case, the best performance was expected to occur when the largest rotation range ( ±180° ) was selected, because a larger range provided a better diversity of phyllotactic orientations. However, the best segmentation performance was achieved with the rotation range of $\pm 135^{{}^{\circ}}$ , which suggests that the selections of rotation angles for individual leaves actually may not be independent events. According to the manual measurements, the phyllotactic angles of leaves had a mean value of 185.1° and a standard deviation of 57.0°. Therefore, given the orientation of a leaf at rank *n*, the potential orientation of the leaf at a higher rank *n*+1 should not be completely random, but should respect a conditional probability distribution *p*(**O**(*rank*=*n*+1)|**O**(*rank*=*n*)) instead. Consequently, the feasible rotation angle for the leaf at rank *n*+1 should obey a conditional probability distribution *p*(*θ*| **O**(*rank*=*n*+1)) as well instead of a uniform distribution *U*(*−θ, θ*). The inaccurate assumptions on the rotation angle distribution may introduce a certain bias towards the expectations. In future work, modifications can be applied to the leaf rotation augmentation to test whether a better performance can be achieved in this manner.

The segmentation with leaf rotation augmentation with respect to the principle axis demonstrated an obvious improvement according to **Table 1**, which supports the hypothesis that leaf orientation difference was one of the sources of dataset diversity. However, leaf orientation changes caused by the spatial utilization and sunlight direction is usually complex. A simple leaf rotation with respect to the principle axis might be insufficient to simulate the real situation and introduce certain unrealistic leaf appearances, especially when dealing with leaves with large curvatures.

#### 3.1.10 Leaf crossover

As for the leaf crossover augmentation, the best performance was obtained with $N_{c}=4$ according to **Table 1**. Given that the average number of leaves per plant was 7.4 in our training set, this shows that the optimal segmentation performance was achieved by exchanging approximately half of the leaves within a plant point cloud. As introduced in Section 2.3.2.3, the leaf crossover operation was usually followed by an online down sampling process. The online down sampling process improved stemwork segmentation *F*1 score to 0.58 (Section 3.1.1), while the leaf crossover augmentation resulted in a stemwork *F*1 score of 0.65 with the optimum $N_{c}$ . This suggests that the leaf crossover operation further improves the stemwork segmentation performance on the basis of online down sampling augmentation.

### 3.2 General discussions

**Figure 10:**
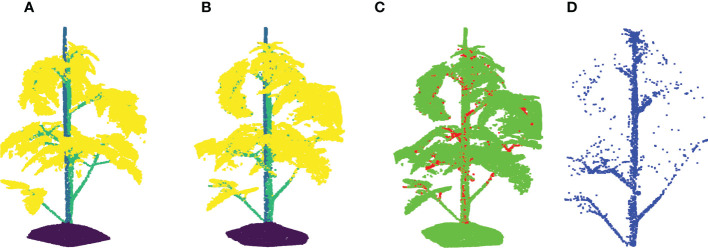
Stemwork segmentation results with leaf crossover augmentation. **(A)** Shows the ground truth labels for individual points; **(B)** Reveals the network predictions of labels; **(C)** Shows the differences between **(A, B)**; **(D)** reveals the extracted stemwork points.

As shown in **Table 1**, all the data augmentation methods achieved certain improvement on the segmentation performance more or less with the optimal parameter selections. Leaf crossover with $N_{c}=4$ resulted in the largest increase in *F*1 score, both on stemwork segmentation as well as on the whole plant segmentation. Comparing with other types of data augmentation methods, leaf crossover enables a larger alteration over the original point cloud, which leads to a wider diversity of the dataset with respect to plant appearance. Meanwhile, leaf crossover is able to mostly maintain the original attributes of the point cloud because the crossover operation is only applied to leaf instances with the same cultivars and ranks. The stemwork segmentation results with leaf crossover augmentation are shown in **Figure 10**.

The data augmentation methods being used in this paper have the potential feasibility to be applied to the 3D plant data other than tomato plants. This requires the target plants to have a similar architecture as the tomato plants being used in the proposed work. Specially for the local data augmentation approaches, the instance labels are always required to localize the plant organs, which may slightly increase the working load during the manual point cloud annotation process. In the proposed work, only one type of data augmentation was used for each experiment of training. One potential direction for future improvement would be the combination strategy of different augmentation methods.

**Figure 11:**
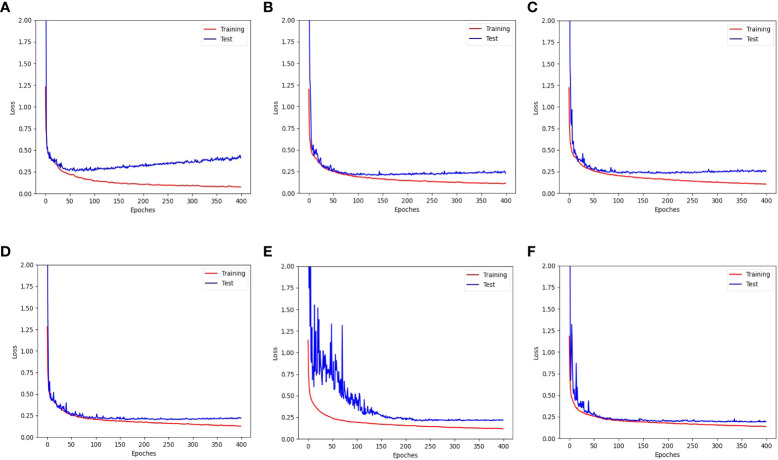
Training and test loss curves obtained with the top five data augmentation methods - **(B)** cropping, **(C)** leaf translation, **(D)** leaf rotation with respect to the vertical axis, **(E)** global jittering, and **(F)** leaf crossover - compared to **(A)** the baseline result without using data augmentation.

**Figure 11** shows the training and test loss curves obtained with the top five data augmentation methods - cropping, leaf translation, leaf rotation with respect to the vertical axis, global jittering, and leaf crossover - compared to the baseline result. A serious overfitting is observed in the train-test loss curves of the baseline result after training for 60 epoches, while the overfitting phenomenon has been significantly improved employing the data augmentations mentioned above. Particularly in the train-test loss curves obtained with leaf rotation with respect to the vertical axis, global jittering, and leaf crossover, the overfitting problem is hardly observed.

**Figure 12:**
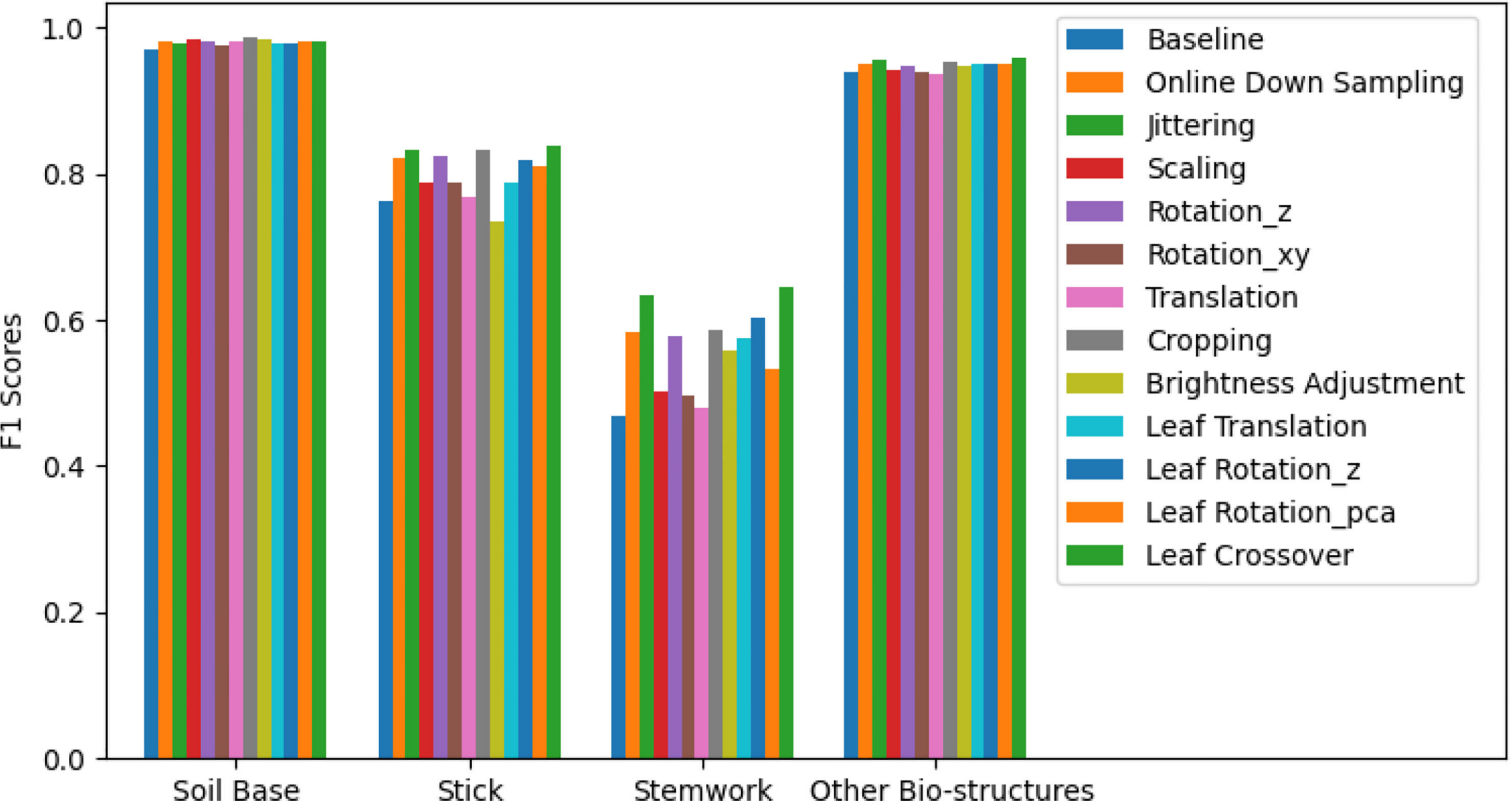
Segmentation *F*1 scores for classes of soil base, stick, stemwork, and other bio-structures respectively. The segmentation performance achieved with individual data augmentation methods was represented by selecting the optimal parameters.

**Figure 13:**
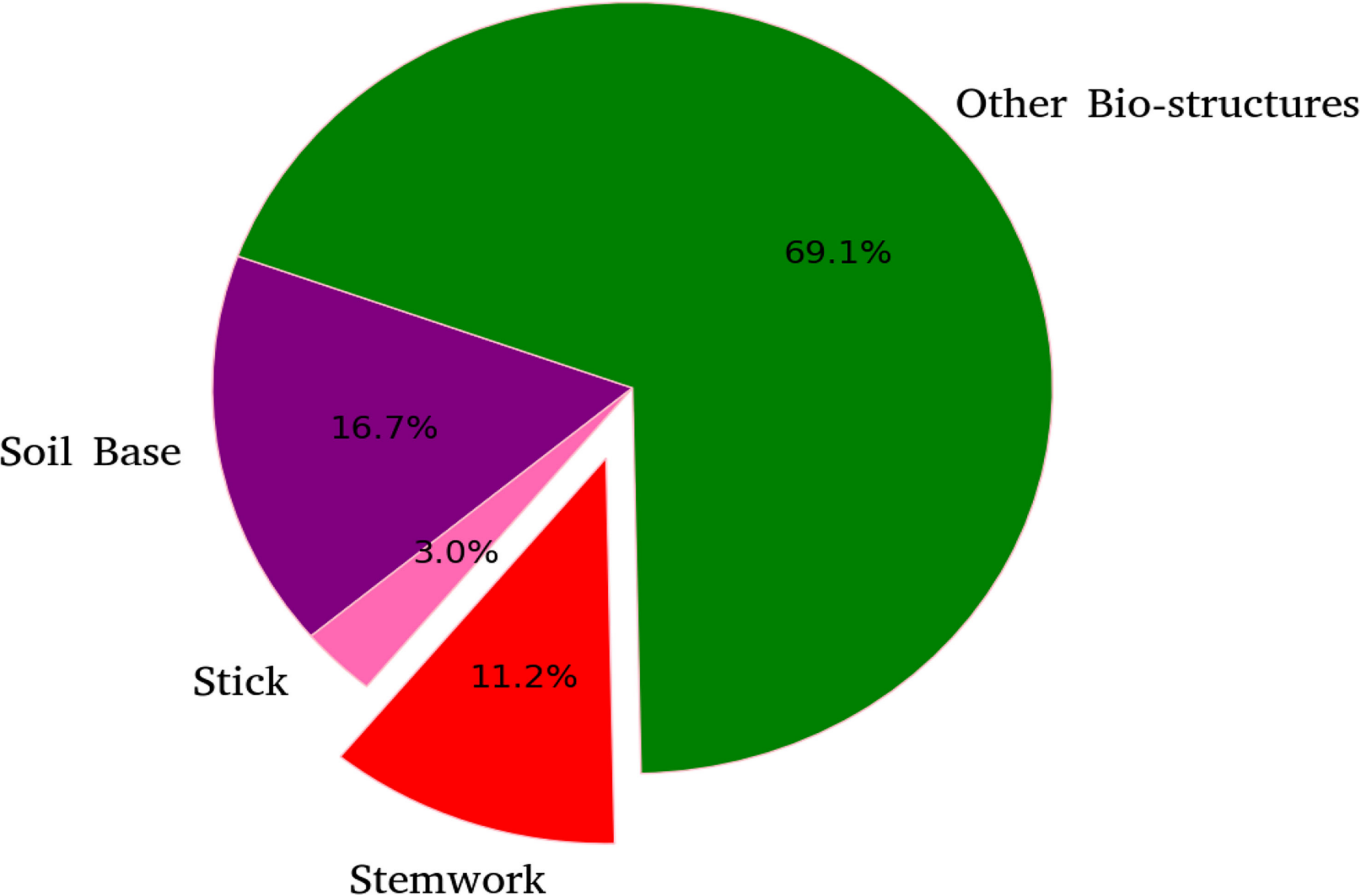
Proportions of points for individual classes with respect to the point clouds in test dataset.

The segmentation *F*1 scores for individual classes (soil base, stick, stemwork, and other bio-structures) achieved by individual data augmentation methods are shown in **Figure 12**. The result presented in **Figure 12** was obtained using the optimal parameters. The segmentation performance of the soil base and other bio-structures was always better than that of the stick and the stemwork. This was mainly caused by the poor separability of the stick class and the stemwork class. One of the factors leading to this poor separability was the similarity in geometrical shape, because both the stick and the stem have a long cylinder-like shape. Combined with the fact that the stick and the stem were usually close together, it was rather tough to separate these two classes from each other. On the other hand, as for petioles, rachis, and petiolules which are also parts of the stemwork, the labeling process contained certain subjective errors. Due to the limitations in point cloud density, stemwork points close to the terminus of leaves or the top of the plant could not be identified even manually, which led to certain stemwork points to be labeled as other bio-structures by mistake. These subjective errors in labels might somehow confuse the deep neuron network during the training process, and result in certain mis-classifications. It can be clearly observed in **Figure 10C** that most of the errors in the predictions of stemwork points occur near the terminus of leaves or the top of the plant, which provides evidence for this view point. Apart from the separability, another reason leading to the poor segmentation performance of stick and stemwork classes was the calculation of loss value. In the original PointNet++ configuration, the loss value was obtained by averaging the cross entropy loss values of individual points. However, in our specific case, a problem of unbalanced number of points within individual classes exists. As shown in **Figure 13**, stick and stemwork points only occupied 3.0 and 11.2% of the total respectively, while most of the points belong to the soil base and other bio-structures. This led to a phenomenon that the network paid more attention to the classes with the majority of points because they have a strong influence on the averaged cross entropy loss, while classes with less points were overlooked to a certain extent. In the same way, the observation that the segmentation improvement for soil and other bio-structures was not significant compared to that of the stick and the stemwork can be explained. According to **Figure 12**, the data augmentation methods achieved a comprehensive improvement on the segmentation performance of all the classes. The segmentation improvement of stick and stemwork class mostly benefited from the corrections of false positives within the classes soil and other bio-structures. However, the number of points being corrected only included a small proportions within the soil and other bio-structures classes, thus the corresponding improvement for these classes was not obvious. In contrast, the points whose predictions were corrected occupied large proportions within classes of stick and stemwork, which resulted in a relatively obvious improvement. This also provides an evidence for us to use stemwork *F*1 scores as the metric to select the optimal parameters instead of the *F*1 scores of other classes or the class-average *F*1 scores. Since the most obvious improvement was found in the segmentation of stemwork class, to use the segmentation performance of other classes or the average performance as the criteria may decrease the separability during the comparison of individual data augmentation methods. This view point can be easily proven through **Table 1** as well.

**Figure 14:**
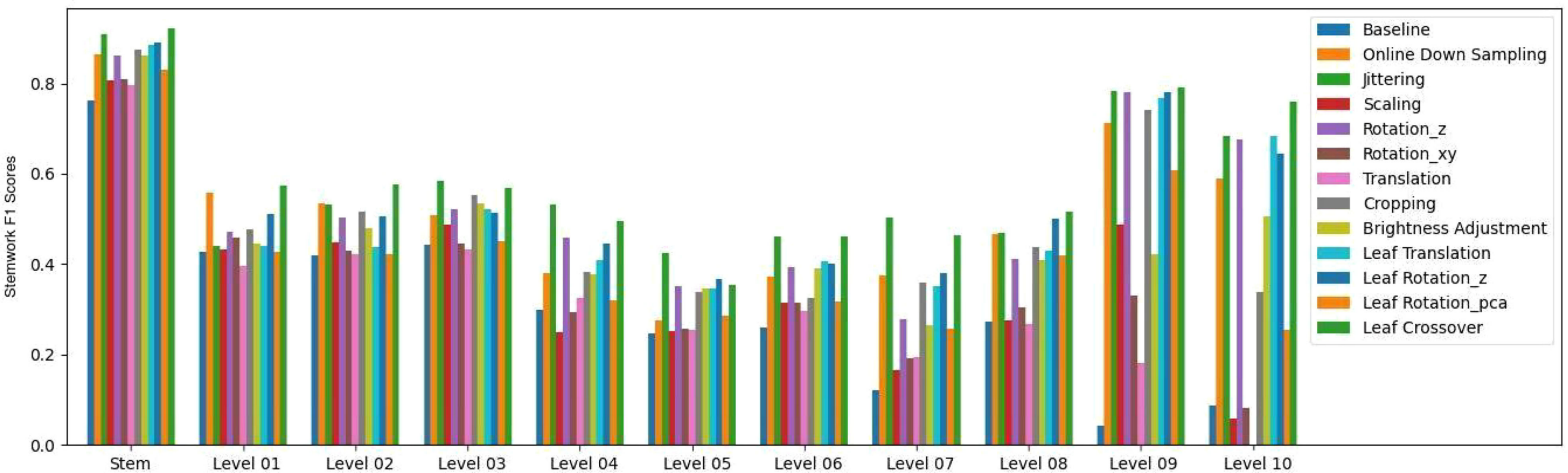
Stemwork segmentation *F*1 scores for the stem part and respective ranks.

The stemwork segmentation *F*1 scores with respect to the stem part and individual ranks are shown in **Figure 14**. The data augmentation methods used in this paper illustrated a clear improvement on the stemwork segmentation performance with respect to the stem part and individual ranks. The stem part generally yielded the highest part-wise *F*1 score, and the *F*1 score gradually decreased when the rank increased. The main reason is that the separability between stemwork and leaflets is getting poorer when the rank increases. On the other hand, the manual labeling may also introduce subjective errors due to the low separability at high ranks, which introduces a certain bias during the training process. It is worthwhile to mention that the performance of rank nine and rank ten was not that representative because in our dataset only one test point cloud out of five contained rank nine and ten.

## 4 Conclusions

Semantic segmentation of plant point clouds is considered a vital research domain for its contributions to modern plant science. In this paper, we looked at 3D segmentation of tomato plants point clouds, and conducted a deep research on potential data augmentation methods aiming to solve overfitting problems caused by the limited size of the training set. Employing PointNet++ as the semantic segmentation backbone, five existing global data augmentation methods were used to enhance the diversity of the dataset. On top of that, we introduced two global augmentation methods (cropping and brightness adjustment) and three local augmentation methods (leaf translation, leaf rotation, and leaf crossover) based on the specific attributes of our tomato plant point cloud dataset. The results show that, when using the optimal parameters, all the data augmentation methods improve the segmentation result. The five data augmentation methods with the best performance were leaf crossover, global jittering, leaf rotation with respect to the vertical axis, leaf translation, and cropping, which demonstrated stemwork segmentation *F*1 score improvements by 0.18, 0.16, 0.15, 0.13, and 0.12 respectively.

Nevertheless, there are some drawbacks of the presented work. First, the test set being used contains only five point clouds and the training set 35 point clouds. Therefore, there still might be certain fluctuations within the test results although we repeated the training for five times and get the average performance for the evaluation. In this case, it was not sufficient either to make further discussions with respect to the cultivars. Knowing that different tomato cultivars maintain different plant structures, an evaluation of augmentation performance with respect to the cultivars is able to provide conclusions on the potential adaptability of each augmentation method over different plant architectures.

## Data availability statement

The raw data supporting the conclusions of this article will be made available by the authors, without undue reservation.

## Author contributions

BX: Original idea, methods, experiments, discussions and paperwork. JS: Methods and experiments. HB: Methods, discussions and paperwork. GK: Original idea, methods, experiments, discussions and paperwork. All authors contributed to the article and approved the submitted version.

## Footnotes

1 A petiole normally refers to the first main internode on a leaf, which is directly connected to the main stem.

2 Rachis refers to all the following internodes after petiole on a leaf. The collection of all the petioles and the rachis internodes forms the secondary internodes of a tomato plant stemwork.

3 Petiolules refer to the tertiary internodes within a tomato plant.

4 Leaves and stem structures commonly have colours close to green, while soil and stick generally show a black colour in our point cloud dataset (**Figure 2A**).

5 The function get_rotation_matrix_from_axis_angle() in the Python library open3d was used: http://www.open3d.org/docs/latest/python_api/open3d.geometry.OrientedBoundingBox.html
